# Research progress on composite nerve guidance conduits with immune-regulatory functions

**DOI:** 10.3389/fimmu.2025.1622508

**Published:** 2025-06-10

**Authors:** Shuxuan Zhang, Xinyue Sun, Xuewa Yang, Yulan Fan, Yuxin Liang, Jiaying Li, Jue Ling

**Affiliations:** ^1^ Key Laboratory of Neuroregeneration of Jiangsu and the Ministry of Education, Co-Innovation Center of Neuroregeneration, Medical School of Nantong University, Nantong University, Nantong, China; ^2^ Healthcare Department, Nantong Third People’s Hospital, Affiliated Nantong Hospital 3 of Nantong University, Nantong, China

**Keywords:** peripheral nerve injury, immunomodulatory, biomaterials, nerve grafts, conduit

## Abstract

Peripheral nerve injury (PNI) has emerged as a critical clinical challenge due to its high disability rate and socioeconomic burden. Traditional autologous nerve grafting, limited by donor shortages and risks of secondary surgeries, has driven tissue-engineered nerve conduits to become a research hotspot. This review systematically summarizes recent advances in immunomodulatory nerve conduits, focusing on the biological properties, degradation mechanisms, and pivotal roles of natural materials (e.g., collagen, chitosan, silk fibroin) and synthetic materials (e.g., poly (lactic-co-glycolic acid) (PLGA), polylactic acid (PLA), and polycaprolactone (PCL) in regulating macrophage polarization. The potential of composite materials to synergistically optimize mechanical performance and bioactivity of nerve conduits is also discussed. Furthermore, this review envisions future trends in nerve conduits, including the integration of 4D printing, smart-responsive systems, and personalized designs to overcome current therapeutic limitations. By integrating multidisciplinary perspectives from materials science, immunology, and regenerative medicine, this review aims to provide innovative theoretical frameworks and technical pathways for efficiently repairing PNI, advancing clinical translation.

## 1 Introduction

### 1.1 Research progress in peripheral nerve injury

Peripheral Nerve Injury (PNI) refers to structural or functional abnormalities in the peripheral nervous system caused by various factors (1, 2), leading to sensory, motor, and autonomic nervous dysfunction (3). These injuries often result from traumatic events such as car accidents, gunshot wounds, sports injuries, and surgical accident (4). Peripheral nerve injury often leads to sensory and motor dysfunction in patients (5). The treatment period following peripheral nerve injury typically exceeds three months (6), accompanied by chronic neuropathic pain, which reduces patients’ quality of life and significantly impacts social productivity (6). Currently, PNI impacts over 20 million people, and the annual medical expenditure for PNI treatment in the United States reaches $150 billion, imposing a huge economic burden on society (4). Due to high treatment costs and suboptimal efficacy, PNI treatment remains a critical issue requiring attention.

**Figure 1:**
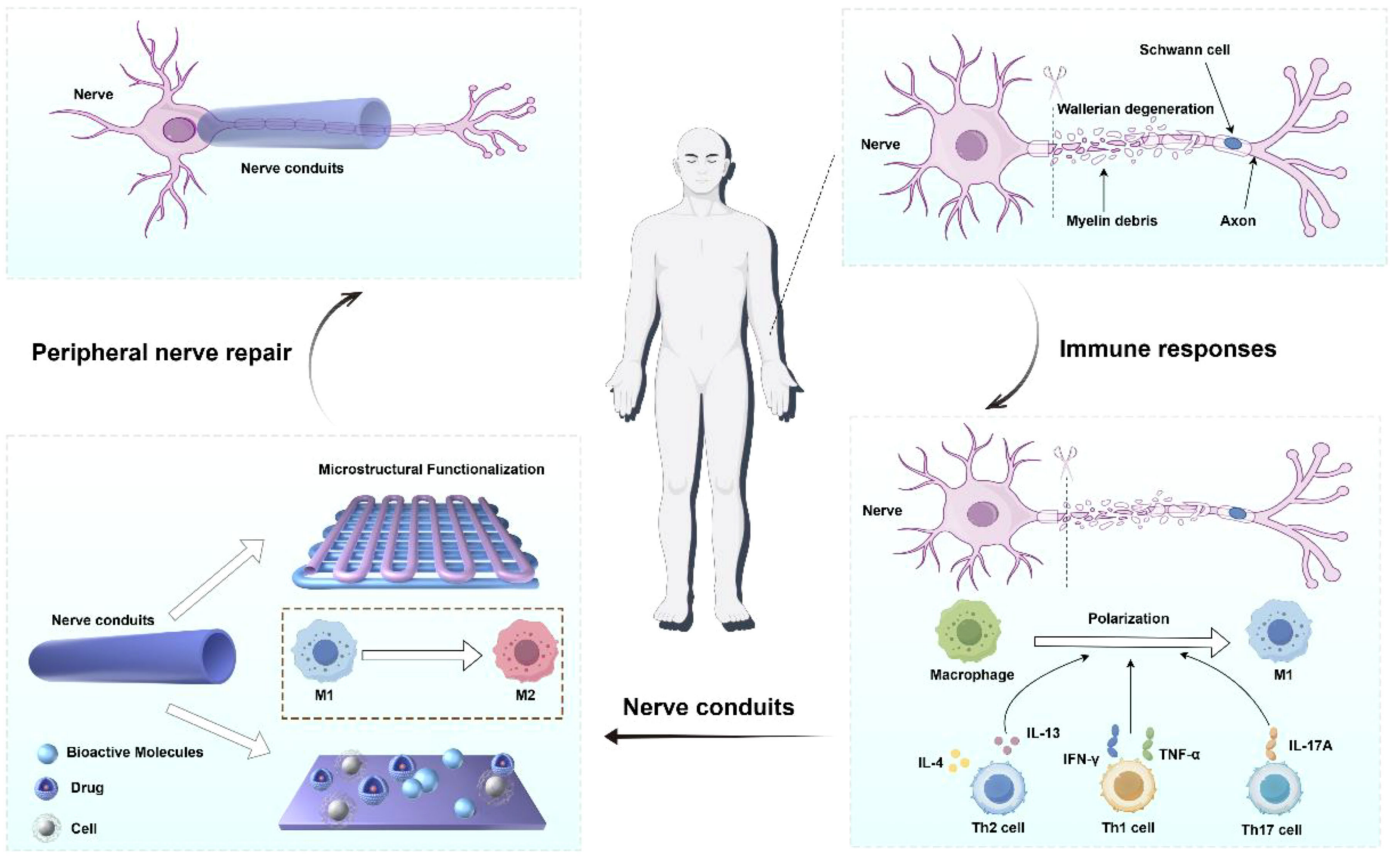
Design of composite nerve guidance conduits with immune-regulatory functions.

Clinical treatment strategies for PNI usually depend on the injury type, severity, etiology, and individual differences (7). Peripheral nerve injuries (PNI) were classified into five grades based on the severity of damage and functional loss. Grades I and II can recover slowly and spontaneously, while Grades III, IV, and V involve injuries to the endoneurial tubes, perineurium, and epineurium, respectively, typically requiring surgical intervention (8, 9). For peripheral nerve atrophy caused by underlying pathologies, pharmacotherapy and physical therapy are commonly employed, though these approaches lack reparative effects on structural defects such as nerve transection (10). To address nerve transection injuries, key techniques for PNI repair and regeneration include: direct neurorrhaphy, autologous nerve grafting, allogeneic nerve transplantation, and bioengineered neural scaffolds (11). For short-distance defects (<3 mm), clinical practice employs nerve anastomosis to suture the nerve (12). The “gold standard” for treating long-distance peripheral nerve defects is autologous nerve grafting (13), where grafts are harvested from other body parts. Optimal outcomes occur when the diameter and axon density of the donor and recipient nerves match (14). Autologous nerves exhibit excellent biocompatibility and support the proliferation of Schwann cells and other cells (15). However, their clinical application is limited by factors such as limited supply, the need for a second surgery, and mismatches between donors and recipients (16). Over the past few decades, researchers have explored biological tubular grafts, such as blood vessels, arteries, muscle fibers, bone conduits, and allogeneic nerve grafts. However, concerns about limited availability, poor mechanical properties, and high morbidity at the donor site have restricted their use (17). Therefore, by integrating multidisciplinary perspectives from materials science, immunology, and regenerative medicine, this review aims to provide innovative theoretical frameworks and technical pathways for efficiently repairing PNI, advancing clinical translation (**Figure 1**).

### 1.2 Immunomodulatory role of nerve regeneration conduits

Over the past few decades, researchers have focused on developing artificial nerve grafts to replace autologous nerve grafts (18). The U.S. Food and Drug Administration (FDA) has approved several nerve conduits made from materials like type I collagen, chitosan, and polyvinyl alcohol for nerve defect repair (19). However, these approved grafts are only suitable for defects shorter than 3.0 cm and ineffective for severe nerve defects (20). A nerve conduit is a tissue-engineered artificial pipeline used to bridge peripheral nerve defects, guiding axonal and neural regeneration and functional recovery by connecting the two ends of the transected nerve (21).

When axons are damaged in PNI, the distal part undergoes axonal and myelin decomposition, a process known as Wallerian degeneration (22). A key event in Wallerian degeneration is the intrinsic degeneration of the injured axon (23). However, PNI is not an isolated event; damaged axons trigger a multicellular response involving multiple components, often accompanied by Schwann cell dedifferentiation and immune response activation (24). Substantial evidence shows that immune cells are recruited to the injury site within hours to days after PNI, playing a critical role in nerve regeneration (25). Macrophages are the most well-studied cell type: they not only remove phospholipid debris and regulate Schwann cell activity but also release numerous axon regeneration factors to promote axonal growth under microenvironmental guidance (26). Macrophages exhibit high plasticity, differentiating into pro-inflammatory (M1) and anti-inflammatory (M2) phenotypes under different stimuli. M1 macrophages activate immune responses to eliminate cells and microbes, while M2 macrophages are immunosuppressive, promoting tissue remodeling and repair (27). After PNI, CD4^+^ Th2 cells also secrete interleukin-4 (IL-4) and interleukin-13 (IL-13), promoting the polarization of M2-type macrophages, releasing neurotrophic factors, and inhibiting inflammatory cytokines. Evidence indicates that M2 polarization promotes peripheral nerve regeneration, unlike M1 polarization (28).

Additionally, T cells play a dual role in the repair of PNI, where their dynamic balance directly influences the regulation of neural regeneration and the immune microenvironment. Following peripheral nerve injury, Th1 cells secrete pro-inflammatory cytokines such as IFN-γ and TNF-α, which activate microglia and disrupt the blood-nerve barrier (BNB), exacerbating neural damage. Th17 cells release interleukin-17A (IL-17A), inducing neuroglial cells to produce pro-inflammatory factors that lead to demyelination and axonal injury. Post-injury, dorsal root ganglion (DRG) neurons secrete chemokines like CXCL13 to recruit CXCR5^+^ CD8^+^ T cells into the neural tissue, establishing a chronic inflammatory microenvironment (29). In contrast, regulatory T cells (Tregs) secrete interleukin-10 (IL-10) and TGF-β, suppressing the activity of Th1/Th17 cells, alleviating neuroinflammation, and promoting the expression of neurotrophic factors to support neuronal survival (30, 31). Therefore, nerve conduit technology provides a novel strategy for nerve repair by utilizing biomaterials to deliver T cells or their regulatory factors to modulate the immune microenvironment.

Nerve conduits physically guide nerve regeneration by providing directional channels for axonal growth, preventing disordered axonal growth and scar tissue invasion, and isolating the regenerating nerve from surrounding fibrosis. They can also create a microenvironment conducive to M2 polarization of macrophages through microstructural design, cytokine loading, and drug release (32, 33). Various nerve conduits have been developed, optimizing mechanical properties, macrostructure, and luminal microstructures to better mimic the natural extracellular matrix (ECM) of neural tissue (34, 35). Additionally, Furthermore, nerve conduits can regulate the immune microenvironment through the incorporation of immunomodulatory agents. For example, conduits loaded with neurotrophic factors (e.g., nerve growth factor, NGF) promote CD4^+^ Th2 polarization, inhibit macrophage M1 phenotype transition, and enhance Treg infiltration. Additionally, nerve conduits loaded with immune-modulating factors (e.g., IL-10) suppress NF-κB activation and reduce the release of pro-inflammatory cytokines (36, 37).

## 2 Material basis of immunomodulatory nerve conduits

### 2.1 Natural materials

Advances in the isolation, purification, and manufacturing of natural biomaterials have enabled the production of robust acellular conduits (38, 39). Natural biomaterials are derived from non-mammalian macromolecules (e.g., chitosan, silk fibroin) or classical components of the mammalian ECM (e.g., collagen, elastin) (40). Due to their biological origin, these materials exhibit high compatibility with human tissues, significantly reducing inflammation, foreign body rejection, and enabling active regulation of the nerve regeneration microenvironment (41). Natural biomaterials have a reduced risk of immune rejection due to their compositional similarity to human tissues (42). Additionally, natural biomaterials possess inherent bioactivity, containing native cell recognition sites (e.g., Arg-Gly-Asp sequences) that promote cell adhesion, proliferation, and differentiation (43). Most importantly, they exhibit controllable degradability, with non-toxic degradation products metabolized via enzymatic or hydrolytic pathways (44). However, conduits fabricated from natural biomaterials often suffer from insufficient mechanical properties to adequately support tissue regeneration. Furthermore, certain materials (e.g., chitosan) may induce chronic inflammation, and since most natural biomaterials are derived from animal sources, they carry a risk of pathogen residue contamination (45). Nonetheless, as “biologically friendly” materials, they provide a basis for promoting M2 polarization of macrophages and offer more physiologically relevant solutions for nerve conduit design.

#### 2.1.1 Collagen

Collagen, a natural polymer widely expressed in all organs and tissues, is easily accessible. Due to its excellent biocompatibility and low immunogenicity, it rarely induces inflammation or rejection after implantation, making it an approved clinical material for nerve conduits (46). Abundant collagen sources and optimized extraction processes meet the high demand for collagen in tissue engineering. Collagen promotes peripheral nerve regeneration: after nerve injury, the expression of various collagens (e.g., type IV and VI collagen) in peripheral nerves is upregulated (47). Type VI collagen has recently been identified as a new regulator of peripheral nerve regeneration; its sustained release enhances M2 polarization of macrophages, promoting neural regeneration and functional recovery after PNI (48). The collagen surface is rich in cell adhesion sites, facilitating Schwann cell migration and secretion of neurotrophic factors to accelerate axonal regeneration (49). Hye Yeong Lee et al. developed a multi-channel nerve conduit with collagen as the matrix using 3D printing and alginate sacrificial template method to form channels. The 9-channel conduit showed better biocompatibility, higher cell proliferation, and superior performance in *in vivo* nerve regeneration, myelin regeneration, inflammation reduction, and angiogenesis compared to other channel numbers (50).

Collagen-based nerve conduits often face issues of insufficient mechanical strength and rapid *in vivo* degradation. Molecular crosslinking strategies are commonly used to improve mechanical strength and stability by reducing α-chain freedom and thermal stability while masking collagenase cleavage sites to enhance anti-enzymatic degradation (51). To address these issues, Chun-Yi Yang et al. prepared a double-layered collagen nanofiber nerve conduit using blow-spinning technology, incorporating chemical crosslinkers to delay degradation. The blow-spun collagen membrane had higher porosity, improving crosslinker permeability. The double-layer conduit promoted Schwann cell growth, neurotrophic factor secretion, axonal regeneration, and functional recovery in rats (52). Type VI collagen plays a key role in promoting macrophage migration and M2 polarization. Lv Dan et al. showed that local delivery of type VI collagen in polycaprolactone (PCL) electrospun conduits significantly increased macrophage recruitment and M2 polarization. Sustained release of type VI collagen in the conduit microenvironment triggered M2 polarization, enhancing nerve regeneration (53).

#### 2.1.2 Chitosan

Chitosan is derived from chitin, a long-chain polymer of N-acetylglucosamine, via deacetylation (54). The degree of deacetylation affects the survival, proliferation, and activity of supportive cells like Schwann cells (55). Chitosan degradation products, chitosan oligosaccharides, are absorbed by the body, stimulate M2 polarization of macrophages, promote Schwann cell proliferation, prevent apoptosis, and exhibit neuroprotective effects during peripheral nerve regeneration (56). Due to its cationic properties, chitosan disrupts the negatively charged membranes of pathogens like *Staphylococcus aureus* and *Escherichia coli*, providing broad-spectrum antibacterial activity and reducing postoperative infection risk, especially in infection-prone scenarios like diabetic peripheral neuropathy (57). Its surface positive charge also enhances adhesion of Schwann cells and neural stem cells, promoting axonal regeneration (58). Chitosan’s solubility increases in acidic environments, allowing design as a smart carrier for pH-responsive release of neurotrophic factors or anti-inflammatory drugs to improve local drug utilization (59).

Chitosan-based nerve conduits often lack mechanical strength; pure chitosan conduits are brittle and prone to fracture due to muscle movement after implantation. Studies show that supplementing chitin or increasing acetylation improves conduit stability (60). Macro- and microstructural designs also significantly enhance mechanical properties. Jiang et al. fabricated a chitosan-based composite multi-channel nerve conduit using warp knitting, consisting of a warp-knitted chitosan scaffold and internal oriented NS-chitosan fibers. The conduit degraded over 90 days, providing sustained protection for peripheral nerve regeneration, and achieved regeneration efficacy close to autologous nerve grafts in a 10 mm nerve defect model (61). In macrophage studies during peripheral nerve regeneration, chitosan conduits often serve as carriers for proteins/factors or bridge defects in gene-knockout mice, providing a material basis for exploring protein/factor roles in macrophage polarization (62).

#### 2.1.3 Silk fibroin

Silk fibroin, a natural polymer extracted from silkworm cocoons, is used in nerve conduit research due to its unique physicochemical and biological properties (63). It is typically obtained by boiling cocoons in 0.5% Na_2_CO_3_, leading to various modified silk fibroins. Silk fibroin exhibits high tensile strength and flexibility; thin-walled conduits made from it do not collapse or deform during long-distance nerve defect repair, showing excellent mechanical properties (64). Tomoki Matsuo et al. prepared an absorbable silk fibroin nerve conduit using a novel freeze-thaw process, with water content >90% and 98.5% shape recovery after 50% compression, demonstrating good structural stability (65).

The degradation rate of silk fibroin can be adjusted via crystallinity to match nerve regeneration speed; its degradation products are amino acids, non-toxic and non-inflammatory, with excellent biocompatibility (66). The silk fibroin surface, rich in β-sheets, mimics the ECM, promoting Schwann cell migration and axonal extension. Its internal porous structure facilitates nutrient diffusion and angiogenesis (67). Silk fibroin can be loaded with neurotrophic factors, stem cells, or anti-scarring drugs to enhance regenerative capacity (68, 69). Commercial silk fibroin conduits like SILKBridge are already available; Olga Politikou et al. reported the first clinical use of SILKBridge for human digital nerve reconstruction, with follow-up showing good recovery, no inflammation, or scarring, confirming its safety, efficiency, and biocompatibility (70).

Wang et al. designed a composite nerve scaffold using regenerated silk fibroin loaded with poly(3,4-ethylenedioxythiophene):polystyrene sulfonate (PEDOT: PSS) and dimethyl fumarate (DMF). The conduit sustained DMF release and used piezoelectric effects to detect sciatic nerve movement, regulating the inflammatory microenvironment. Combined with electrical stimulation, it promoted M1 to M2 polarization of macrophages by inhibiting Schwann cell apoptosis and reducing inflammatory factor release, supporting peripheral nerve regeneration and functional recovery (71).

### 2.2 Synthetic materials

Synthetic material-based nerve conduits are a research hotspot for repairing peripheral nerve defects, offering core advantages in customizable structure and function (72). Synthetic biomaterials can regulate mechanical properties (e.g., stiffness, porosity) and degradation rates through chemical modifications, and synthetic polymers exhibit minimal batch-to-batch variation, making them suitable for standardized production. Widely used 3D printing substrates like polylactic acid (PLA) and polycaprolactone (PCL) can form biomimetic microchannels or oriented nanofibers via 3D printing or electrospinning, mimicking ECM topological structures to guide Schwann cell migration and axonal orientation (73). Adjusting the molecular weight of polymers like poly (lactic-co-glycolic acid) (PLGA) or the ratio of lactic to glycolic acid in PLGA can synchronize degradation rate with nerve regeneration, avoiding secondary surgery (74). Integrating conductive materials or loading neurotrophic factors endows synthetic conduits with biochemical signals and electrical stimulation (75). However, pure synthetic materials lack bioactivity, often requiring additional modification for cell adhesion (76), and suffer from mechanical mismatch issues: PLA has high elastic modulus causing local stress concentration. The degradation byproducts of synthetic biomaterials may trigger inflammatory responses. For instance, materials like PLGA (poly(lactic-co-glycolic acid)) generate acidic degradation products during breakdown, leading to a local decrease in pH that exacerbates macrophage infiltration and inflammatory reactions. The implantation of polyethylene glycol (PEG) hydrogels may activate macrophages, leading to fibrosis and fibrous capsule formation. Studies have shown that the material’s surface charge, porosity, and degradation products collectively influence the behavior of immune cells (77). Thus, the long-term safety of such materials *in vivo* still necessitates comprehensive evaluation.

#### 2.2.1 Poly (lactic-co-glycolic acid)

PLGA, an artificial copolymer synthesized via ring-opening polymerization of lactic and glycolic acids, is FDA-approved for drug delivery due to its bioinertness and mechanical strength, widely studied in regenerative medicine (78). However, acidic degradation products may limit its use in tissue engineering. Lu et al. found that *in vitro* PLGA scaffold degradation caused significant local acidification, with higher PLGA content correlating with worse nerve regeneration and increased macrophage infiltration during degradation (79). Despite this, its excellent degradability allows precise control of degradation rate via monomer ratio adjustment, avoiding long-term foreign body retention. It can also form microspheres to load neurotrophic factors for sustained release, enhancing the bioactivity of the regenerative microenvironment. PLGA’s processability enables use in electrospinning and 3D printing. Pure PLGA conduits have 40% fewer Schwann cells than natural materials; in long-distance injuries, insufficient vascularization affects regeneration, so vascular endothelial growth factor A (VEGF-A) is often introduced to promote angiogenesis. Huang et al. used PLGA as the outer layer of a conduit, transfected Schwann cells with VEGF-A, and loaded them in methacryloyl gelatin to evaluate peripheral nerve repair. Transfected Schwann cells provided a stable VEGF-A source, promoting angiogenesis (80). Panjian Lu et al. injected type I and IV collagen into chitosan/PLGA composite scaffolds, finding that type I collagen recruited more M2 macrophages, while type IV recruited more M1 macrophages, suggesting M2 macrophages induced by type I collagen may aid repair, whereas M1 macrophages induced by type IV collagen may be harmful (81).

#### 2.2.2 Polylactic acid and polycaprolactone

PLA is considered as an excellent nerve conduit substrate due to its biocompatibility and absorbability. As an aliphatic polyester, it has been widely studied and applied in nerve regeneration, featuring low allergenicity, low toxicity, and high biocompatibility (82). Similar to PLGA, PLA’s degradation rate can be precisely adjusted via molecular weight to match needs for repairing injury. As a common 3D printing material, it is abundant and low-cost. However, the highly hydrophobic surface limits its bioactivity, requiring functionalization for cell adhesion. Cardoso et al. studied the effect of inosine on nerve regeneration using PLA conduits for sciatic nerve transection, showing inosine significantly enhanced regeneration and functional recovery (83).

PCL, similar to PLA, has low immunogenicity but better flexibility and lower elastic modulus, matching natural nerve mechanics more closely. Its longer degradation cycle makes it suitable for supporting long-segment defects, but it may cause foreign body reactions, often requiring secondary surgery (84). Li et al. prepared PCL conduits with spiral melt-spun fibers on the outer surface and aligned electrospun fibers on the inner surface, finding that aligned inner nanofibers effectively promoted nerve regeneration, while spiral fibers provided anti-kinking and compression resistance, showing clinical potential (85). Zhan et al. loaded single-layer graphene (SLG) and nanodiamond (ND) into polycaprolactone (PCL) fibers to construct an anisotropic nerve guidance conduit (SLG/ND/PCL NGC) with nanogrooves and aligned fibers. The conduit promotes directional growth and myelination of Schwann cells by activating the Piezo1 signaling pathway, triggering Ca^2^ influx, thereby activating NFAT and Krox-20 molecules to regulate myelin-related gene expression. In a rat sciatic nerve defect model, SLG/ND/PCL NGC significantly improved nerve conduction velocity, the number of myelinated axons, and muscle function recovery, with effects comparable to autologous nerve grafting (86).

#### 2.2.3 Polypyrrole

Peripheral nerves, composed of sensory and motor neurons, are electrically sensitive tissues, making external electrical stimulation (ES) an effective method for promoting regeneration (87). To facilitate ES, conductive materials like polypyrrole (PPy) have been developed for tissue engineering. PPy, with conductivity of 10^1^-10^3^ S/cm, promotes Schwann cell migration and axonal orientation via ES, but its poor solubility and non-degradability limit applications (88, 89). It is often copolymerized with degradable materials to form hybrid systems. Zhao et al. prepared a PPy/silk fibroin conductive composite scaffold, which, with ES, activated MAPKs signaling to promote *in vivo* axonal and myelin regeneration (90). Tian et al. developed a novel delivery system using layered carbon nanotubes, PPy, dexamethasone, and nerve growth factor, which inhibited LPS-induced inflammatory cytokine secretion under controlled ES, showed no toxicity to PC12 cells and primary neurons, and promoted cell adhesion, growth, and neurite outgrowth (91).

### 2.3 Composite materials

While natural materials offer excellent cell affinity and regenerative microenvironments, they often have low mechanical strength, uncontrollable degradation, and batch variability limiting clinical use. Synthetic materials, though customizable in mechanics and degradation, lack bioactive signals and their degradation products may acidify the microenvironment. Composite materials combine natural and synthetic materials to leverage synergistic effects, overcoming single-material limitations by integrating mechanical properties, bioactivity, conductivity, and controllable degradation to mimic the complex microenvironment of nerve regeneration (92). Composite strategies can create smart systems with biomimetic topology, dynamic degradation matching, and functional expansion, avoiding inherent defects like immunogenicity and poor mechanics (93). However, composites face challenges such as complex processing, poor interfacial compatibility, high production costs, and structural instability due to differential degradation rates. Nano-additives (e.g., graphene) and crosslinkers (e.g., glutaraldehyde) may also pose biocompatibility risks, potentially causing cytotoxicity or immune reactions (94).

Liu et al. prepared PLCL/SF conduits combined with NGF-loaded conductive TA-PPy-RGD hydrogel via electrospinning. The RGD-modified tannic acid (TA)-polypyrrole (PPy) hydrogel provided cell adhesion sites, a conductive microenvironment, and sustained NGF release, promoting nerve cell proliferation. Compared to traditional and composite conduits, PLCL/SF/NGF@TA-PPy-RGD showed superior nerve regeneration due to its innovative combination of biocompatibility, bioactivity, and conductivity, highlighting the need for continued research for clinical translation (34). Cheng et al. constructed a PPy-coated PCL/silk fibroin scaffold, finding that ES promoted nerve regeneration and M2 polarization of macrophages, with *in vitro* ES significantly enhancing M2 pro-regenerative polarization. Bioinformatics analysis revealed differences in signal transducer and activator of transcription expression related to M2 gene promotion, indicating ES’s impact on macrophage phenotype (95).

## 3 Immunomodulatory strategies for nerve conduit fillers

### 3.1 Functionalization with bioactive molecules

Nerve conduits, as critical supportive structures, guide and promote nerve regeneration. Functionalizing internal fillers, especially with bioactive molecules, is a key strategy to enhance repair efficacy (96, 97). Bioactive molecules include neurotrophic factors, cytokines, and growth factors, all playing vital roles in nerve repair (98). Loading these factors into hydrogels within conduits can significantly improve the microenvironment, regulate immune responses and inflammation, and promote axonal growth (99).

Neurotrophic factors like nerve growth factor (NGF), brain-derived neurotrophic factor (BDNF), and glial cell-derived neurotrophic factor (GDNF) are widely used in filler functionalization (100–102). They bind to neuronal surface receptors, activate downstream pathways, and promote axonal extension. Especially in the early injury stage, they enhance neuron growth, inhibit apoptosis, and improve functional recovery (103). Hydrogels, as carriers, provide a suitable microenvironment for regeneration and enable sustained factor release via controlled-release systems, maintaining long-term bioactivity.

Growth factors like vascular endothelial growth factor (VEGF) and fibroblast growth factor (FGF) are crucial for improving blood supply and angiogenesis. After nerve injury, blood supply significantly affects regeneration; VEGF promotes vascular regeneration in the injury area, providing oxygen and nutrients to support nerve growth (104). Combined with hydrogel carriers, growth factors achieve long-term stability.

Cytokines like transforming growth factor β (TGF-β) and interleukin-10 (IL-10) regulate the immune microenvironment during nerve repair (105, 106). They inhibit excessive immune inflammation, reduce fibrosis, and promote nerve regeneration and angiogenesis. Loading cytokines into hydrogels creates a regenerative-friendly immune microenvironment, avoiding adverse immune factors (107).

The bioactive molecule functionalization strategy for hydrogel fillers in nerve conduits provides multi-level support for nerve repair, promoting neuron growth, survival, and differentiation while regulating immunity, inflammation, and the local microenvironment for long-term effective recovery (108) Yang et al. first established a pro-inflammatory model to validate the guiding and repairing role of M2 macrophages in long-distance PNI, then developed a self-assembling biomimetic peptide hydrogel scaffold encapsulating M2-derived regenerative cytokines and extracellular vesicles. This scaffold mimicked the immune microenvironment mildly, remodeling the local environment for M2 polarization and recruitment, facilitating long-distance peripheral nerve regeneration (109).

### 3.2 Microstructural functionalization

Microstructural functionalization of nerve conduit fillers is a key strategy for promoting nerve repair (37). Fillers must provide physical support and mimic the ECM microenvironment to guide neuron growth, differentiation, and axonal extension. Microstructures can be designed by adjusting porosity, fiber alignment, and surface properties to mimic the natural ECM, promoting nerve regeneration (110).

Porosity and pore size distribution are critical for repair efficacy. A reasonable pore structure provides space for cell adhesion, axonal extension, and angiogenesis (111). Techniques like gas foaming, solvent casting, and 3D printing enable precise control of porosity and pore size to optimize filler performance (112).

Incorporating nanostructures significantly enhances physiological functions of fillers (113). Nanofibers, particles, and meshes mimic the 3D network of natural ECM, providing an ideal microenvironment for nerve regeneration (114). Electrospun nanofibers, in particular, form collagen-like networks within conduits, offering adhesion sites for neurons and guiding axonal orientation via physical cues (115). Studies show that surface topography can influence macrophage phenotype by adjusting cytoskeleton: M2 macrophages are typically more elongated than M1 (116). Oriented electrospun nanofibers can regulate macrophage morphology to an elongated state, promoting M2 polarization and reducing pro-inflammatory factor secretion (117). Small surface pores also reduce inflammatory stimulation of macrophages (118).

Multilevel structural design combines micro- and macro-scale features for better repair effects (119). At the macroscale, ordered pores facilitate cell migration; at the microscale, grooved or oriented nanofibers guide neuron directionality (120). Multilevel structures act synergistically at different scales to enhance regeneration.

Sun et al. inspired by the ordered distribution of nerve fibers and endogenous electric fields, studied the synergistic effect of ES and topological cues on the immune microenvironment during peripheral nerve regeneration. They fabricated a nerve conduit with oriented electrospun nanofibers using a polyurethane copolymer containing conductive aniline trimer and degradable L-lysine. *In vitro* results showed the conduit promoted macrophage recruitment, induced M2 polarization, and enhanced Schwann cell migration and myelination. In a rat sciatic nerve injury model, it increased M2 macrophage proportion and improved regeneration, demonstrating potential applications from an immunomodulatory perspective (121).

Furthermore, accumulating evidence has elucidated the regulatory role of material stiffness in modulating innate immune cell responses. Mechanistic studies demonstrate that elevated substrate stiffness tends to promote macrophage polarization toward pro-inflammatory (M1) phenotypes, while uncoated high-stiffness substrates paradoxically induce anti-inflammatory (M2) polarization. Neutrophils exhibit reduced migration velocity yet enhanced spreading on rigid matrices, potentially exacerbating inflammatory responses through enhanced extracellular trap formation. Notably, natural killer (NK) cells leverage mechanosensing capabilities via surface stiffness receptors, with rigid substrates shown to upregulate cytotoxic markers and potentiate target cell elimination (122, 123). Fang et al. developed an electroconductive multiscale nerve conduit featuring multilayered stiffness gradients through architectural engineering (32). This design achieved coordinated macrophage repolarization toward M2 phenotypes via stiffness-dependent mechanotransduction. Additionally, aligned microfibers with stiffness gradients served as topographical guidance cues, facilitating directed migration of vascular endothelial cells and macrophages to accelerate neovascularization, thereby establishing metabolic support systems for functional regeneration.

### 3.3 Controlled drug release

Effective and sustained drug delivery is crucial for nerve regeneration. Nerve conduits, as supportive structures, can incorporate controlled-release systems in fillers to maintain local drug concentration and enhance repair (124). Controlled drug release has become a key functionalization strategy, enabling sustained, timed, and quantitative drug release to maximize efficacy (125).

Design of controlled-release systems relies on various materials and mechanisms, including hydrogel-based sustained-release systems (126), nanocarriers (127), and self-responsive release systems (128). Hydrogels, with good biocompatibility and adjustable degradation, are ideal carriers: crosslink density, hydrophilicity, and pore structure can be tuned for precise release control (129). They maintain a moist microenvironment and stable long-term drug release, ensuring sustained action on nerves. Anti-inflammatory drugs, for example, are released gradually via diffusion or hydrolysis, promoting repair (130).

Nanotechnology offers new approaches for controlled release: nanoparticles (e.g., lipid, polymer nanoparticles) efficiently load and target drugs, preventing degradation or rapid loss. Particle size, surface properties, and composition can be adjusted for precise release (92, 131). As reported by Wan et al., a nerve conduit featuring a dynamic 3D interconnected porous network was developed by integrating chitosan with multifunctional gelatin microcapsules. These gelatin microcapsules served as drug carriers and demonstrated efficient adsorption of positively charged insulin-like growth factor-1 (IGF-1) through electrostatic interactions. The degradation-controlled release mechanism of gelatin effectively regulated drug delivery kinetics, preventing both localized drug overdosage and premature depletion. The study revealed that IGF-1 synergized with the 3D porous architecture to promote macrophage polarization toward the M2 phenotype via activation of the PI3K/Akt signaling pathway. This immunomodulatory effect established an anti-inflammatory microenvironment, which significantly enhanced the regeneration of sciatic nerve defects in rat models (132).

Self-responsive systems, another emerging direction, adjust release rates based on microenvironmental changes (pH, temperature, electric field) during nerve repair. As pH and temperature change with regeneration, self-responsive hydrogels release drugs adaptively, optimizing efficacy (133, 134).

Multi-drug co-delivery is also promising: combining neurotrophic factors (135), antioxidants (136), etc., addresses the complex biology of nerve repair. Controlled-release systems enable synergistic release of multiple drugs at different rates and time windows, ensuring stage-specific effects. Dexamethasone, a common immunosuppressive small molecule, is widely used in implantable materials for its low cost and availability. Kumar et al. found silk hydrogels sustained release of dexamethasone and IL-4, promoting M2 polarization and maintaining tissue function (137).

Yu et al. regulated zein-induced immune responses by adjusting the size, pore structure, and dexamethasone loading of zein microspheres, inhibiting neutrophil recruitment and promoting M2 polarization via pore structure design. They then prepared high-porosity zein conduits loaded with zein microspheres for bridging 15 mm sciatic nerve defects in rats, showing better repair efficacy at the degradation stage, with porous microspheres enabling sustained drug release to enhance tissue repair (138).

### 3.4 Cell loading

The core goal of cell loading in nerve conduit fillers is to introduce cells or cell-derived materials that directly support nerve repair. Unlike traditional drug loading, cell loading participates in repair via multiple mechanisms: promoting neuron growth/differentiation, regulating the local microenvironment, and enhancing neural network reconstruction (139). Stem cell loading is a key direction, with mesenchymal stem cells (MSCs) (140) and induced pluripotent stem cells (iPSCs) (141) being promising due to their self-renewal and differentiation potential into neurons or glial cells. These cells differentiate into neural tissue at the injury site, secrete neurotrophic/growth factors, and promote repair (142). Glial cell loading, especially astrocytes, is another strategy. They secrete growth/nutritional factors and ECM, support neuron growth, participate in local immunity, clear cellular debris, and maintain microenvironmental stability, accelerating healing and axonal regeneration (143). Fibroblast loading provides structural support via collagen/ECM secretion, forming a regenerative matrix and releasing local growth factors to facilitate repair, improving conduit stability and microenvironment (144, 145). Yuan et al. inspired by spinal cord anatomy, developed a multi-channel fibrous conduit with multicellular distribution for spinal cord injury repair. Using directional freeze-casting, they created a conduit with hierarchical parallel channels and oriented layered structures, inoculating MSCs in central channels and Schwann cells in peripheral channels. *In vitro*, cell interactions promoted Schwann cell migration, MSC differentiation, endothelial cell angiogenesis/migration, and M2 polarization of macrophages. *In vivo*, it reduced glial scarring, promoted neuron regeneration, myelination, and functional recovery in rats (146).

## 4 Conclusions and future perspectives

Multidisciplinary collaboration and technological innovation can drive breakthroughs in nerve conduit fabrication, aiming to surpass autologous nerve grafts and provide efficient, intelligent clinical solutions. Future nerve conduits will develop toward multifunctional composite scaffolds integrating biomaterials, drug delivery systems, cell loading, and dynamic regulation mechanisms. These conduits will not only provide physical support but also integrate neurotrophic and immunomodulatory factors to dynamically respond to biological needs during repair. Material selection will focus on biocompatibility, degradability, mechanical properties, and microstructural optimization, combined with smart design to adapt to changes at the injury site over time. With technological maturity, clinical translation should emphasize standardized performance evaluation systems, including biological assessment, mechanical testing, and drug release detection, to ensure optimal clinical potential. Future designs will be more personalized, tailored to patient-specific injury types and locations using modern imaging for precise implantation via image navigation. Nerve conduits will also evolve toward intelligence, personalization, and multifunctionality, integrating 4D printing, smart drug delivery, and organoid technology to enhance nerve repair and muscle function recovery. Clinical translation will become smoother with standardized evaluation, bringing more effective treatments to PNI patients.
